# Some clarifications regarding statistical inference

**DOI:** 10.1111/aogs.14414

**Published:** 2022-09-15

**Authors:** Andrew V. Frane

**Affiliations:** ^1^ Department of Psychology Occidental College Los Angeles California USA

Sir,

The commentary “Current controversies: null hypothesis significance testing”^1^ is largely correct, but contains two statements that require clarification.

**FIGURE 1:**
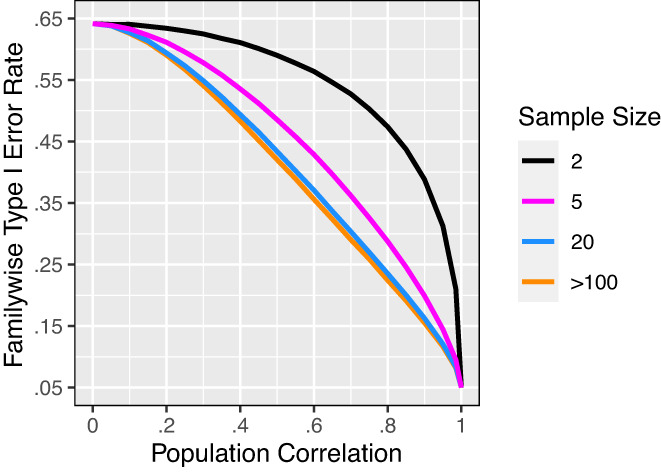
Familywise type I error rate (probability of at least one type I error) for 20 two‐sided *t* tests as a function of homogeneous population correlation among outcome variables (based on 10^6^ simulations for each of 22 correlation values, using multivariate‐normal outcome variables, all true null hypotheses, and *α* = 0.05)

First, the commentary stated, “when 20 hypothesis tests are performed the probability of a type I error is at least 0.64.” On the contrary, for 20 valid tests at the 0.05 level, probability of a type I error is *as high as* approximately 0.64. Indeed, familywise probability of type I error is maximized when all outcomes are independent (notwithstanding negative dependence) and all null hypotheses are true.^2^ And by a well‐known formula, that maximum probability is 1 − (1–0.05)^20^ ≈ 0.64 (where 0.05 is the alpha level for each test and 20 is the number of true null hypotheses). Probability of a type I error will be lower than that maximum—though still potentially egregious—if outcomes are positively correlated (Figure 1) or if any null hypotheses are false (since false null hypotheses cannot produce type I errors).^2^

The commentary also stated, “Studies with large sample sizes are important because as sample size approaches the population size, the sample estimates have increased accuracy when estimating the population parameters.” Large samples are indeed important, and they do tend to make parameter estimates more accurate—but mainly because larger sample sizes provide more data, not because they approach the population size.^3^ One might assume that as sample size increases, it necessarily “approaches the population size.” But in the vocabulary of statistical inference, the *population* is the entire set of individuals about whom inferences are made. Thus, for most experiments in medicine, population size is theoretically infinite, or at least too large to be meaningfully “approached.” For example, in a clinical trial to evaluate a treatment for an illness, the population of interest presumably includes everyone who could potentially ever have the illness—even individuals who have not been born yet.

Even in special circumstances where the population is finite, population size is typically irrelevant to estimation precision (notwithstanding highly exceptional cases).^3^ A sample size of 200 certainly tends to provide better estimates (smaller standard errors) than a sample size of 100. But as statisticians have shown,^3^ a sample size of 100 does not tend to provide substantially better estimates when it comes from a population of 2000 than when it comes from a population of 2 billion. In fact, precision is 0.025% higher in the latter case (if estimating a mean difference between randomized groups).^4^ Thus, the inferential benefits of large samples are generally due to the sheer amount of data, not the proximity of population size to sample size.^3^

The clarifications in this letter may seem minor to some. But they are important because they involve fundamental statistical concepts, and one statistical misunderstanding often leads to another. For example, one article that is cited in the commentary made the same statement about larger sample sizes “approaching” population size—and then incorrectly concluded that the type I error rate of each test decreases as sample size increases.^5^ To avoid confusion, I recommend that the commentary be corrected.
